# Morality in the echo chamber: The relationship between belief in COVID-19 conspiracy theories and public health support and the mediating role of moral identity and morality-as-cooperation across 67 countries

**DOI:** 10.1371/journal.pone.0273172

**Published:** 2022-09-07

**Authors:** Theofilos Gkinopoulos, Christian Truelsen Elbæk, Panagiotis Mitkidis

**Affiliations:** 1 Department of Philosophy and Social Studies, University of Crete, Crete, Greece; 2 Department of Management, Aarhus University, Aarhus, Denmark; University of Padova, ITALY

## Abstract

Believing in conspiracy theories is a major problem, especially in the face of a pandemic, as these constitute a significant obstacle to public health policies, like the use of masks and vaccination. Indeed, during the COVID-19 pandemic, several ungrounded explanations regarding the origin of the virus or the effects of vaccinations have been rising, leading to vaccination hesitancy or refusal which poses as a threat to public health. Recent studies have shown that in the core of conspiracy theories lies a moral evaluation component; one that triggers a moral reasoning which reinforces the conspiracy itself. To gain a better understanding of how conspiracy beliefs about COVID-19 affect public health containment behaviors and policy support via morality-relevant variables, we analysed comprehensive data from the International Collaboration on the Social & Moral Psychology (ICSMP) of COVID-19, consisting of 49.965 participants across 67 countries. We particularly explored the mediating role of two levels of morality: individual and group-based morality. Results show that believing in conspiracy theories reduces adoption of containment health-related behaviors and policy support of public health measures, but moral identity and morality-as-cooperation significantly mediate this relationship. This means that beliefs in conspiracy theories do not simply constitute antecedents of cognitive biases or failures, nor maladaptive behaviors based on personality traits, but are morally infused and should be dealt as such. Based on our findings, we further discuss the psychological, moral, and political implications of endorsement of conspiracy theories in the era of the pandemic.

## Introduction

Since December 2019, COVID-19 has been affecting societies and countries across the globe, posing important questions for individual and social lives amidst a hygienic crisis with multifaceted implications. While the virus was rapidly spreading around the world, conspiracy theories and other forms of fake news were finding a fertile ground to spread too. From the very beginning of the pandemic, the Director General of the World Health Organization (WHO) claimed that beyond the battle of the virus itself, WHO is also battling the spread of conspiracy theories regarding the virus and any misinformation that undermines the importance of urgent and necessary measures to tackle the infection rates [1]. Indeed, several explanations of the origin of the virus were circulated during the beginning of the pandemic, based on conspiracies such as that COVID-19 had been intentionally engineered as a political mode of controlling the masses, or as a hoax which had been spread out, worldwide, by 5G networks. These conspiracy theories led in their turn to further hoaxes, creating vaccination hesitancy and denial threatening to become an immense public health problem [2].

### Moral identity and morality-as-cooperation

As it was previously mentioned, morality in the era of the pandemic has been prominent and is worth of further empirical investigation. In our multi-country study, we conceptualized morality as an individual trait, as well as a cooperative group-relevant variable.

To begin with, moral identity, reflecting the individualized conceptualization of morality, refers to the subjective importance of morality to someone’s personal identity, typically represented as a trait-based difference between people or as a trait in the form of self-schema [3]. Moral identity is composed by a private “having” component (internalization) and a public “doing” component (symbolization). Internalization concerns the degree of centrality of moral traits for someone’s self-concept, while symbolization has more to do with the degree of reflection of these moral traits in public action and choices taking place in certain social settings [4]. From a motivational perspective, internalization relates to seeking for maintaining self-consistency between identity and behavior. On the other hand, symbolization is motivated by goals of self-presentation and recognition of the self in terms of a social entity that seeks for self-affirming feedback from other people [5]. To date, research mainly focuses on the internalization aspect, calling for more empirical attention to the symbolization–“doing”–aspect of moral identity [5,6].

Concluding, symbolization and internalization act as synergetic in people’s moral reasoning and decision making. Additionally, Colby and Damon [7] suggested that what differentiates moral people from other people is the extent to which they perceive their own sense of morality and personal goals as united. Extending this argument, Frimer and Walker [8] proposed a reconciliation model, which relies on moral identity and describes moral identity as entailing agency-related motives (e.g. self-interest) and communion-related motives (e.g. other-oriented morality, cooperation, prosocial behavior). Such motives are supposed to act as synergetic and not as competing and, eventually, the integration of personal and communion moral goals provide a powerful source of commitment to action.

Morality-as-cooperation, in this context, is exemplified in community-based and societal challenges, where moral actions are needed. Morality-as-cooperation, indeed, identifies problems and societal challenges, which can be solved via exhibiting cooperative behavior, such as reciprocation or help towards the group [9]. In light of mutualism as a prerequisite for morality-as-cooperation behaviors [10], there are situations, which beg for cooperative behaviors and where individuals benefit each other by jointly and cooperatively working with others. Coordinating to achieve a mutual advantageous outcome requests joint coalitions and efforts to compete with threats and uncertainties [11].

### Conspiracy theories and moral concerns

During the pandemic, adherence to health protective behaviors and compliance with public health policies has become a morally expected issue with relevant moral concerns, when public health protection rules are violated [12]. Prioritizing public health and responding to public health risks also raised multiple moral dilemmas that have become the subject of extensive debates [13]. To this end, we aimed to consider morality-relevant variables in empirical investigation of the topical debate on conspiracy theories that have flourished during the pandemic and the degree to which morality variables mediate the relationship between endorsement of COVID-19 conspiracy theories and adherence to public health behaviors and support for equivalent policies.

Conspiracy theories share a common theme, in the way that they rely on negative and traumatic events conceptualized and explained as intentional and plotted plans from powerful elites against populations [14]. These “hidden plans” often entail a morality component. Indeed, Leone et al. [15] posit that conspiracy theories entail a moral evaluation component, on the grounds that traumatic and frightful events originate from malevolent elites [16] and, thus, trigger a moral reasoning relevant to the actual understanding of conspiracy theories [17]. Farinelli [18] has described conspiracy theories as “morality tales based on archetypal narratives about right versus wrong, good versus evil” (p.4). This conceptualization of conspiracy theories can reflect the degree to which people, who are attentive to morality issues may also believe in conspiracy theories which, in turn, appeal to people’s moral perceptions, values and judgements.

Moral concerns may be relevant to people’s individual and also collective well-being, referring to individual and group-foundations respectively [19]. Moral foundations concerning people’s group membership relate more strongly with endorsement of conspiracy beliefs than moral foundations concerning people’s individual moral traits and judgements [15]. This claim lies on the nature of conspiracy beliefs per se as a reflection of an “us-versus-them” mentality. This mentality is often anchored in events against nations, religious, and other large groups as victims of such conspiracy theories [20] that are supposed to attack the populations. Leone et al. [15] have suggested that the group perspective of conspiracy theories activates a group identity perspective when people think in terms of their binding to the group and not in terms of individual binding. However, following the aforementioned conceptualizations of moral identity and morality-as-cooperation, especially in light of the Reconciliation Model, as dimensions that can complement each other, we hypothesize that there will be a significant positive association between both morality-as-cooperation and moral identity with conspiracy beliefs about COVID-19 (H1a). The threats that are entailed in conspiracy theories are supposed to trigger a defensive way of life on the part of the people, reflected in their moral principles of self-responsibility and cooperation to overcome the impact of such threats. This explains the expected positive direction of the expected association.

### Morality, beliefs and conspiracy theories and health protective behaviors

While several conspiracy-based explanations of various events can fascinate people across time and be harmless [21], most conspiracy theories relevant to public health crises can be dangerous and harmful for individual and societal well-being and resilience [22,23]. These types of conspiracy theories include misinformation and disinformation related to COVID-19 and cast doubt on the existence of the virus per se, in turn making people who endorse such conspiracy theories less likely to comply with public health measures [24].

Concerning moral foundations, health choices, and practices of protection of public health during the pandemic exemplify a choice of individuals to depend on their moral attitude to protect themselves and others. Containing pandemic-related behaviors as actions that people need to take to tackle the spread of the virus, morality is one key factor that plays an important role in determining the extent to which people will engage, or not, in such actions. Indeed, individuals are supposed to be motivated to take actions, depending on their willingness and intention to defend and protect sacred values, such as their quality of life and health which, in turn, are threatened [25]. People, who endorse conspiracy theories rarely make health-related choices recommended by authorities. Pummerer et al. [26] confirm findings according to which believing in conspiracy theories decreases governmental support and adoption of health-related containing behaviors such as physical distancing. Uscinski and Parent [27] have showed that people high in conspiracy beliefs were less likely to engage in cooperative and altruistic behaviors or donation practices. More recent findings come from a study by Imhoff and Lamberty [28] on conspiracy worldviews of COVID-19 and engagement with pandemic preventive and hygienic behaviors. In this study, conspiracy theories that described the COVID-19 pandemic in terms of a hoax, were more strongly associated with reduced containment pandemic-related behavior (e.g., increased hygiene behaviors, handwashing, physical distance maintenance). This finding was observed in the United Kingdom and the United States.

Further, it has been suggested that when morality is central to people’s self-concept, then moral judgements of actions, such as intention to adopt hygienic measures, are translated into actual behavior [29]; here, scholars have shown that moral judgement and empathy for significant others is a key predictor of engagement with preventive pandemic-related behaviors. Additionally, Pagliaro et al. [30] have evidenced that discretional COVID-19 related behaviors are exhibited when they are prescribed by their very moral essence to foster the collective well-being and welfare of the community amidst the pandemic crisis. We, thus, hypothesize that conspiracy beliefs will be negatively associated with pandemic-related behaviors and policy support, but this association will be positively mediated by moral identity and morality-as-cooperation (H1b).

Overall, with our study we aim to show how both political and non-political underpinnings of conspiracy theories about COVID-19 can lead to public health containment behaviors and support of measures. The contribution of our study lies on identifying the associations between conspiracy beliefs and health-related behaviors and policy support via two levels of morality: individual and group-based morality, examining how such associations occur across 67 countries that differ in the way they implemented measures and contained the spread of the virus. Up until now, very few studies take both the individual and group dimension of morality at the same into account [15,31].

### Rationale of the study

In the present study, we used morality-as-cooperation to measure the cooperative component of morality that ties individuals to their living community. Additionally, we used a trait-based measure of moral identity [32] to discover the traits that compose people’s unique moral identity as part of individuals’ self-concept rather than their cooperation with community group members. Moral identity is conceptualized as an individual trait-based tendency to consider morality as central to individual self-concept and sense of self-consistency on moral action and personal identity [5]. Formation of moral identity is, thus, linked with individual characteristics and contexts for moral actions [33]. When individuals integrate morality-relevant values into their self-concept, then a moral self-identity arises [34]. On the other hand, morality-as-cooperation conceptualizes morality in terms of a group-focused behavior and cultural solutions to issues of cooperation and conflict that may occur in people’s social life. Morality-as-cooperation is based on interactions between groups and people, characterized by mutual coordination, social exchanges or division and disputes [9,35]. Morality-as-cooperation mechanism motivates people towards altruistic and cooperative behaviors, as well as opportunities to evaluate behaviors of others [36]. To the extent that moral identity can be conceptualized as a foundation for comprehending moral agency in groups and organizations [37], we suggest that moral identity and morality-as-cooperation complement each other. Moral identity, by definition, entails an interactive, cooperative component too, since individuals with moral identity develop a sense of personal responsibility, considering themselves responsible for their actions in particular contexts. Therefore, they act either proactively or reactively and always in congruence between their judgement of the context and their actions for the benefit of themselves and others, who are present in a situation at hand [38].

## Materials and methods

### Participants

In April 2020, a research team from New York University launched a call for participation in a survey, using various social media to collect data across the globe on psychological and social factors that might be related to responses to COVID-19 pandemic, with public health support as the key outcome. We firstly created a survey in English (see supplementary material) and then we sent it to each team for back translation. The survey secured ethical approval from the ethics board at the University of Kent and was conducted in accordance with the Code of Professional Ethics of the British Psychological Society, the Hellenic Psychological Society, the Danish Psychological Association, and with the 1964 Helsinki declaration and its later amendments or comparable ethical standards. Written informed consent was obtained from all individual participants included in the study (see, supplementary material: https://osf.io/y7ckt/). Multi-level models were used for dataset collection and analysis. We report how sample size was determined, all data excluded from the analysis (if any), all manipulations, and all measures in our survey.

For the purpose of this study, we used data collected by an international team of scholars as part of the International Collaboration on the Social & Moral Psychology of COVID-19 project (see https://icsmp-covid19.netlify.app/about.html). Each team was allowed to include additional items after the main survey under their own institutional protocol. Each research team across 67 countries was assigned to collect data from a number of at least 500 participants, a sample which should be representative with respect to gender and age, in their own country or territory, but not all countries achieved a recruitment of representative samples. Out of the 67 countries joined the project, representative samples were recruited in 28 countries, convenience samples were recruited in 36 countries, and both types of sampling were recruited in 3 countries. All authors of this study were a part of this international collaboration.

A sample of N = 51,404 individuals overall across 69 countries participated in our survey. Following the inclusion criteria, participants needed to be 18 years and older, and give informed consent. Raw data obtained from all research collaborators were cleaned, in order to exclude any duplicate answers, as well as those participants, who were younger than 18 years or older than 100 years. Next, we excluded data from two participants from Puerto Rico and 313 participants recruited from the UAE, where it was difficult to establish nationality of participants. This resulted in a total sample of 51,089 participants. For the subsequent analyses of this paper, we also excluded participants who had missing data on all the key variables of our interest. Therefore, we were left with (N = 49,965) participants from 67 countries, where 50.9% were females, 44% males, 0.3% others, and 4.8% unreported. Participants were over 18 years of age and younger than 100 years of age (M = 43.8, SD = 16.05). Countries from all continents (except for Antarctica) took part in our study. Some are overrepresented (e.g., Europe, Americas) while others are underrepresented (e.g., Africa, Middle East). For further information about the distribution of education, country of residence and other demographics across 67 countries, see the complete dataset in our supplementary material here: https://osf.io/tfsza/.

All the analyses reported in our paper were repeated after controlling for differences in methods of sample recruitment. In our analysis, no coefficients that differed as a function of sampling procedure were encountered, which would compromise and/or alter the reported main effects.

### Measures

We used three measures of public health support [31]. We used a *Spatial Limiting Distancing* 5-item scale (Cronbach’s α = 0.74), to measure people’s maintenance of spatial distance and reduction of physical contact, with items such as “During the days of the coronavirus (COVID-19) pandemic, I have been staying at home as much as practically possible”. Furthermore, we used a *Physical Hygiene* 5-item scale (Cronbach’s α = 0.72), to measure people’s adoption of health-related behaviors and improvement of their physical hygiene, with items such as “During the days of the coronavirus (COVID-19) pandemic, I have been washing my hands longer than usual”. Finally, we used a *Policy Support* 5-item scale (Cronbach’s α = 0.81), to measure people’s support towards implementation of health-protective policies and measure as responses to the pandemic, with items such as “During the days of the coronavirus (COVID-19) pandemic, I have been in favor of closing all schools and universities”. We used an 11-point “slider scale with three labels: 0 = “*strongly disagree”*, 50 = *“neither agree nor disagree”*, 100 = *“strongly agree”*. These labels were re-coded to a scale ranged from 0 (strongly disagree) to 10 (strongly agree).

Additionally, we used a 4-item *COVID-19 Conspiracy Beliefs* scale (Cronbach’s α = 0.79), to measure people’s endorsement of conspiracy theories about the origin and the causes of the pandemic, with items such as “The coronavirus (COVID-19) is a bioweapon engineered by scientists.” As it was mentioned in the introduction, in this scale, we included not only political-related items, but also items relevant to the scientific community, interest social groups not necessarily political, as well as global economic issues. A scale from 0 (strongly disagree) to 10 (strongly agree) was used. Engaging government, science, interest social groups, global institutions and an explanation of the virus as a hoax in measurement of conspiracy beliefs helped us avoid effects of political attitudes and ideological preferences on the associations we examine.

Lastly, we measured morality using two scales: First, we used a 7-item *Morality-as-Cooperation* scale [9] (Cronbach’s α = 0.77), to measure different cooperative moral behaviors such as helping groups. Participants were instructed to think when they decide whether something is right or wrong, to rate the extent to which a number of considerations is relevant to their thinking. Example consideration was “Whether or not someone worked to unite a community”. A scale from 0 (strongly disagree) to 10 (strongly agree) was used.

Next, we used a 10-item *Moral Identity* scale [32], to measure people’s self-identification based on moral prosocial attributes. This scale consisted of two subscales; moral identity symbolization and moral identity internalization. Participants first read 9 characteristics (e.g. caring, compassionate etc.) that might describe a person, who could be themselves or it could be someone else. Next, participants had to visualize in their mind the kind of person who has these characteristics. Participants were asked to imagine how that person would think, feel, and act. When they had a clear image of what this person would be like, participants were asked to rate statements such as “I am actively involved in activities that communicate to others that I have these characteristics” (symbolization subscale; Cronbach’s alpha = 0.75) and “Being someone who has these characteristics is an important part of who I am” (internalization subscale; Cronbach’s alpha = 0.68) (see Appendix A, for the full list of items in symbolization and internalization dimensions). A scale from 0 (strongly disagree) to 10 (strongly agree) was used.

Recent research which has also used this data and originated from the same international collaboration project (ICSMP) have used advanced machine-learning algorithms, established measurement equivalence of the moral identity internalization and symbolization sub-scales across the 67 countries included in the data set [39]. The original paper of Van Bavel et al. [31] reported a two-factor model (Internalization and Symbolization), with acceptable internal consistency. This two-factor structure was confirmed in our subsequent machine-learning analysis [39] after correlating residuals of items 8 and 9, and 4 and 7. (CFI = 0.939, RMSEA = 0.077, 95% CI [0.070, 0.084], SRMR = 0.067, Cronbach’s alpha_internalization_ = 0.68, Cronbach’s alpha_symbolization_ = 0.75).

### Covariates

In all analyses, we controlled for: (a) participants’ age, gender, employment status and living area (urban-suburban or rural); (b) participants’ levels of national identification, measured with two items (I identify as [nationality]; Being a [nationality] is an important reflection of who I am.); (c) participants’ political ideology, measured in one item, where participants had to rate their political views in a scale from 0 to 10, where 0 indicated very left-leaning views and 10 indicated very right-leaning views. The full list of scales, along with the dataset in SPSS and a CSV form can be found here: https://osf.io/y7ckt/.

## Results

We initially performed a Principal Components Analysis (PCA) with varimax rotation on the four items measuring Belief in Conspiracy Theories, in order to check whether all items loaded on the same factor. The results of this analysis suggested that all items were captured by one dimension in 9 out of 13 estimation algorithms (Bentler, Optimal coordinates, Acceleration factor, Parallel analysis, Kaiser criterion, SE Scree, R2, VSS complexity 1, Velicer’s MAP) hence allowing us to aggregate the measure of Belief in Conspiracy Theories into one variable (Cronbach’s *α* = .92), used in the subsequent modelling. Summary statistics of all variables included in the analysis are shown in **Table 1**.

**Table 1:** Summary statistics.

	Mean	SD	Median
Age	43.8	16.05	41.00
Spatial Limiting Distancing	7.89	1.87	8.38
Physical Hygiene	7.93	1.89	8.40
Policy Support	7.87	2.27	8.60
Belief in Conspiracy Theories	3.09	2.94	2.50
Morality-as-Cooperation	6.53	1.65	6.57
Moral Identity (Symbolization)	5.68	1.42	5.60
Moral Identity (Internalization)	5.25	1.71	5.20

Next, we fitted two Linear Mixed Effects models (estimated using Restricted Maximum Likelihood) [40] to predict our three dependent variables of Spatial Limiting Distancing, Physical Hygiene and Policy Support. Specifically, in model 1 we predicted our measure of Spatial Limiting Distancing by Belief in Conspiracy Theories, Moral Identity (Symbolization), Moral Identity (Internalization) and Moral-as-Cooperation, with country as a random effect. In model 2 we predicted our measure of Physical Hygiene by Belief in Conspiracy Theories, Moral Identity (Symbolization), Moral Identity (Internalization) and Moral-as-Cooperation again with country as a random effect. Finally, in model 3 we predicted our measure of Policy Support by Belief in Conspiracy Theories, Moral Identity (Symbolization), Moral Identity (Internalization) and Morality-as-Cooperation with country as a random effect, as it was previously done. For all models, all standardized parameters were obtained by fitting the model on a standardized version of the dataset and 95% Confidence Intervals (CIs) and *p*-values were computed using the Wald approximation. Results are reported in **Table 2**.

**Table 2:** Linear mixed effects models.

		Spatial Limiting Distancing			Physical Hygiene			Policy Support	
*Predictors*	*Estimates*	*Std*.*Beta* *(95%CI)*	*p*	*Estimates*	*Std*.*Beta* *(95%CI)*	*p*	*Estimates*	*Std*.*Beta* *(95%CI)*	*p*
Intercept	5.97	0.15 (0.07–0.23)	**<0.001**	6.13	0.13 (0.05–0.21)	**<0.001**	7.32	0.17 (0.07–0.27)	**<0.001**
Belief in Conspiracy Theories	-0.10	-0.13 (-0.14 –-0.12)	**<0.001**	-0.18	-0.12 (-0.13 –-0.11)	**<0.001**	-0.38	-0.22 (-0.23 –-0.21)	**<0.001**
Moral Identity (Symbolization)	0.29	0.13 (0.12–0.14)	**<0.001**	0.22	0.16 (0.15–0.18)	**<0.001**	0.15	0.11 (0.10–0.12)	**<0.001**
Moral Identity (Internalization)	0.09	0.09 (0.08–0.10)	**<0.001**	0.03	0.06 (0.05–0.07)	**<0.001**	0.04	0.02 (0.01–0.03)	**<0.001**
Morality-as-Cooperation	0.20	0.12 (0.11–0.13)	**<0.001**	0.13	0.14 (0.13–0.15)	**<0.001**	0.12	0.12 (0.11–0.13)	**<0.001**
**Random Effects**									
σ^2^	2.98			2.93			3.96		
τ_00_	0.34country			0.38country			0.89country		
ICC	0.15			0.12			0.18		
N	67countries			67countries			67countries		
Observations	49965			49965			49965		
Marginal R^2^ / Conditional R^2^	0.085/0.198			0.080/0.186			0.069/0.240		
AIC	177072.191			176178.144			189783.213		

For model 1, multi-level modelling showed that Spatial Limiting Distancing had a significant negative association with Belief in Conspiracy Theories, while having significant positive associations with Moral Identity (Symbolization), Moral Identity (Internalization) and Morality-as-Cooperation. The intercept model was found to be at 5.97 (95% CI [5.84, 6.29], *t*(44930) = 60.11, *p* < .001). The random effects Intraclass Correlation Coefficient (ICC) for the model indicated that most of the variance in the model was explained by the fixed variables, while 12% of the variance could be explained by the random-effect of country, indicating moderate differences between the 67 countries in the data.

In a similar vein, for model 2, multi-level modelling showed that abidance to Physical Hygiene had a significant negative association with Belief in Conspiracy Theories, while having significant positive associations with Moral Identity (Symbolization), Moral Identity (Internalization) and Morality-as-Cooperation. The intercept for the model was found to be at 6.13 (95% CI [5.93, 6.32], *t*(44930) = 61.97, *p* < .001). The random effects Intraclass Correlation Coefficient (ICC) for the model indicated that most of the variance in the model was explained by the fixed variables, while 12% of the variance could be explained by the random-effect of country, indicating moderate differences between the 67 countries in the data.

For model 3, results showed that Policy Support aimed to combat the spread of COVID-19 had a significant negative association with Belief in Conspiracy Theories and significant positive association with Moral Identity (Symbolization), Moral Identity (Internalization) and Morality-as-Cooperation. The intercept for the model was found to be at 7.32 (95% CI [7.05, 7.59], *t*(44936) = 53.30, *p* < .001) and the random effects ICC for the model indicated that most of the variance in the model was explained by the predictive variables. The model suggests greater variance in the relationship between our predictor variables and the support for policies aimed to combat COVID-19. Specifically, the ICC indicates that 18% of the variance observed in the model could be explained by the random-effect of country, indicating notable differences between the 67 included countries.

Building on the results obtained in the linear mixed effects models, we subsequently fitted three multi-level mediation models in order to test if Moral Identity (Symbolization and Internalization) and Morality-as-Cooperation mediated the negative relationship between Belief in Conspiracy Theories and Spatial Limiting Distancing, Physical Hygiene or Policy support, respectively. The results of these three models are reported in **Tables 3**, **4 and 5**, respectively.

**Table 3:** Multilevel Mediation Analysis, model 1 (Spatial Limiting Distancing).

Parameter	Coefficient	LL (95% CI)	HL (95% CI)	z	p	Label
Belief in Conspiracy Theories → Spatial Limiting Distancing	-0.053	-0.071	-0.028	-5.018	< .001	c
Belief in Conspiracy Theories → Morality-as-Cooperation	0.092	0.074	0.110	10.173	< .001	a_1_
Belief in Conspiracy Theories → Moral Identity Symbolization	0.085	0.067	0.104	10.166	< .001	a_2_
Belief in Conspiracy Theories → Moral Identity Internalization	0.079	0.061	0.101	10.161	< .001	a_3_
Morality-as-Cooperation → Spatial Limiting Distancing	0.138	0.129	0.141	16.321	< .001	b_1_
Moral Identity Symbolization → Spatial Limiting Distancing	0.125	0.119	0.131	16.308	< .001	b_2_
Moral Identity Internalization → Spatial Limiting Distancing	0.119	0.116	0.122	16.304	< .001	b_3_
a_1_b_1_: = a_1_*b_1_	0.012	0.010	0.014	7.821	< .001	a_1_b_1_
a_2_b_2_: = a_2_*b_2_	0.010	0.009	0.011	7.612	< .001	a_2_b_2_
a_3_b_3_: = a_3_*b_3_	0.009	0.008	0.010	7.548	< .001	a_3_b_3_
total: = c + (a_1_*b_1_)	-0.041	-0.061	-0.021	-3.792	< .001	total
total: = c + (a_2_*b_2_)	-0.043	-0.063	-0.023	-3.801	< .001	total
total: = c + (a_3_*b_3_)	-0.044	-0.064	-0.024	-3.810	< .001	total

Note: *CI* = Confidence Interval, *LL* = Lower Limit, *HL* = Higher Limit, *p* = p-value.

**Table 4:** Multilevel Mediation Analysis, model 2 (Physical Hygiene).

Parameter	Coefficient	LL (95% CI)	HL (95% CI)	z	p	Label
Belief in Conspiracy Theories → Physical Hygiene	-0.062	-0.087	-0.025	-5.046	< .001	c
Belief in Conspiracy Theories → Morality-as-Cooperation	0.092	0.074	0.110	10.173	< .001	a_1_
Belief in Conspiracy Theories → Moral Identity Symbolization	0.085	0.067	0.104	10.166	< .001	a_2_
Belief in Conspiracy Theories → Moral Identity Internalization	0.079	0.061	0.101	10.161	< .001	a_3_
Morality-as-Cooperation → Physical Hygiene	0.149	0.140	0.152	16.330	< .001	b_1_
Moral Identity Symbolization → Physical Hygiene	0.115	0.109	0.121	16.297	< .001	b_2_
Moral Identity Internalization → Physical Hygiene	0.122	0.119	0.125	16.309	< .001	b_3_
a_1_b_1_: = a_1_*b_1_	0.013	0.012	0.014	7.810	< .001	a_1_b_1_
a_2_b_2_: = a_2_*b_2_	0.009	0.008	0.010	7.654	< .001	a_2_b_2_
a_3_b_3_: = a_3_*b_3_	0.010	0.009	0.011	7.587	< .001	a_3_b_3_
total: = c + (a_1_*b_1_)	-0.049	-0.029	-0.069	-3.779	< .001	total
total: = c + (a_2_*b_2_)	-0.053	-0.043	-0.063	-3.798	< .001	total
total: = c + (a_3_*b_3_)	-0.054	-0.044	-0.064	-3.801	< .001	total

Note: *CI* = Confidence Interval, *LL* = Lower Limit, *HL* = Higher Limit, *p* = p-value.

**Table 5:** Multilevel Mediation Analysis, model 3 (Policy Support).

Parameter	Coefficient	LL (95% CI)	HL (95% CI)	z	p	Label
Belief in Conspiracy Theories → Policy Support	-0.133	-0.188	-0.079	-4.809	< .001	c
Belief in Conspiracy Theories → Morality-as-Cooperation	0.092	0.074	0.110	10.173	< .001	a_1_
Belief in Conspiracy Theories → Moral Identity Symbolization	0.085	0.067	0.104	10.166	< .001	a_2_
Belief in Conspiracy Theories → Moral Identity Internalization	0.079	0.061	0.101	10.161	< .001	a_3_
Morality-as-Cooperation → Policy Support	0.199	0.140	0.211	16.344	< .001	b_1_
Moral Identity Symbolization → Policy Support	0.145	0.139	0.151	16.289	< .001	b_2_
Moral Identity Internalization → Policy Support	0.132	0.129	0.135	16.301	< .001	b_3_
a_1_b_1_: = a_1_*b_1_	0.018	0.016	0.020	7.790	< .001	a_1_b_1_
a_2_b_2_: = a_2_*b_2_	0.012	0.011	0.013	7.682	< .001	a_2_b_2_
a_3_b_3_: = a_3_*b_3_	0.010	0.009	0.011	7.601	< .001	a_3_b_3_
total: = c + (a_1_*b_1_)	-0.115	-0.105	-0.125	-3.821	< .001	total
total: = c + (a_2_*b_2_)	-0.012	-0.002	-0.022	-3.755	< .001	total
total: = c + (a_3_*b_3_)	-0.010	-0.001	-0.020	-3.743	< .001	total

Note: *CI* = Confidence Interval, *LL* = Lower Limit, *HL* = Higher Limit, *p* = p-value.

For multilevel mediation model 1, the model with Spatial Limiting Distancing as a dependent variable was significantly different from a baseline model (Chi2(7) = 27914.77, p < .001) and GFI (.99 > .95), AGFI (.97 > .90) and RFI (1.00 > .90) suggested a satisfactory fit. As in the linear mixed effects model, the results suggest that Belief in Conspiracy Theories is negatively associated with Spatial Limiting Distancing (*b* = -0.053, *z* = -5.018, *p* = < 0.001), but that this relationship is positively mediated by Moral Identity (Symbolization) (*b* = 0.010, *z* = 7.612, *p* = < 0.001), Moral Identity (Internalization) (*b* = 0.009, *z* = 3.548, *p* = < 0.001) and Morality-as-Cooperation (*b* = 0.012, *z* = 7.821, *p* = < 0.001), resulting in a smaller total effect of Belief in Conspiracy Theories on Spatial Limiting Distancing, when such mediations are present. Hence, the results indicate that Moral Identity (Symbolization and Internalization) and Morality-as-Cooperation positively mediates the relationship between Belief in Conspiracy Theories and abidance to an increase in Spatial Limiting Distancing as a result of COVID-19. Results are summarized in Table 3 below.

Next, for multilevel mediation model 2, the model with Physical Hygiene as a dependent variable was significantly different from a baseline model (Chi2(7) = 27921.80, p < .001) and GFI (.99 > .95), AGFI (.97 > .90) and RFI (1.00 > .90) suggested a satisfactory fit. As in the linear mixed effects model, the results suggest that belief in conspiracy theories is negatively associated with Physical Hygiene (*b* = -0.062, *z* = -5.046, *p* = < 0.001), but that this relationship is positively mediated by Moral Identity (Symbolization) (*b* = 0.009, *z* = 7.654, *p* = < 0.001), Moral Identity (Internalization) (*b* = 0.010, *z* = 7.587, *p* = < 0.001) and Morality-as-Cooperation (*b* = 0.013, *z* = 7.810, *p* = < 0.001), resulting in a smaller total effect of Belief in Conspiracy Theories on Physical Hygiene, when such mediations are present. Hence, the results indicate that Moral Identity (Symbolization and Internalization) and Morality-as-Cooperation positively mediates the relationship between Belief in Conspiracy Theories and abidance to an increase in Physical Hygiene as a result of COVID-19. Results are summarized in Table 4 below.

Finally, for multilevel mediation model 3, the model with Policy Support as a dependent variable was significantly different from a baseline model (Chi2(7) = 27936.14, p < .001) and GFI (.99 > .95), AGFI (.97 > .90) and RFI (1.00 > .90) suggested a satisfactory fit. As with mediation model 1, the results suggest that Belief in Conspiracy Theories is negatively associated with Policy Support (*b* = -0.133, *z* = -4.809, *p* = < 0.001), but that this relationship is positively mediated by Moral Identity (Symbolization) (*b* = 0.012, *z* = 7.682, *p* = < 0.001), Moral Identity (Internalization) (*b* = 0.010, *z* = 7.601, *p* = < 0.001) and Morality-as-Cooperation (*b* = 0.018, *z* = 7.790, *p* = < 0.001), resulting in a smaller total effect of Belief in Conspiracy Theories on Policy Support, when such mediations are present. Hence, the results indicate that Moral Identity (Symbolization and Internalization) and Morality-as-Cooperation positively mediates the relationship between Belief in Conspiracy Theories and Policy Support of initiatives aimed at restricting COVID-19 transmission. Results are summarized in Table 5 below.

## Discussion

The aim of this paper was to investigate (a) how Beliefs in Conspiracy Theories led to adoption of health-related behaviors and support of public health policies; (b) how these associations were mediated by Moral Identity and Morality-as-Cooperation. Our findings showed that Beliefs in Conspiracy Theories reduced Policy Support of governmental measures (e.g. lockdowns), as well as the abidance to health-related behaviors (e.g. social distancing, handwashing, mask-wearing etc.) related to the pandemic. Importantly, this relationship was mediated by Moral Identity and Morality-as-Cooperation. While evidence suggests that conspiracy theories exemplify a kind of calling cards that signal the generation of collective action and other political actions [41,42] in the era of COVID-19, it has been shown that collectively adopting health-related behaviors as a form of action, as well as policy support toward the government measures, is reduced when people endorse conspiracy theories about COVID-19 [26,28].

Furthermore, our results confirmed the expected positive association between Beliefs in Conspiracy Theories and Moral Identity (Symbolization and Internalization) and Morality-as-Cooperation [15,20]. Mediation of Moral Identity and Morality-as-Cooperation in the relationship between Beliefs in Conspiracy Theories and containment of health-related behaviors, adds to current findings which have mainly focused on endorsement of moral principles of care and fairness that increase, in turn, people’s inclination to trust the science and government demonstrating higher Policy Support and adoption of prescribed health-related behaviors [30].

Specifically, we have built on and expanded these findings by simultaneously testing Moral Identity as an individual trait and Morality-as-Cooperation as a variable related to collective life, identifying potential similarities or differences in their effects on the adoption of health-related behaviors and Policy Support. Although the theory suggested that Morality-as-Cooperation would have a stronger effect than Moral Identity [15], we found that both Moral Identity and Morality-as -Cooperation both significantly mediated the relationship between Beliefs in Conspiracy Theories and the dependent variables. People’s moral judgements constitute key antecedents of the adoption of containment behaviors and compliance with and support of political decisions to tackle the spread of the virus, but when Beliefs in Conspiracy Theories are high, it is reflected in people’s distrust toward science and government, exhibiting low adoption of relevant preventive hygienic behaviors [43]. (Non) conspiracy explanations of the virus can potentially raise a collective action dilemma. While cooperation between people and their moral foundations could potentially be increased amidst the pandemic, the dilemmatic nature of the underpinnings of the virus–whether the virus is a hoax or it is a fact–seems to determine people’s actual hygienic behaviors and their moral judgements toward each other and themselves, evaluating their position in this context. Similar dilemmas for collective action have been identified in other cases, such as the antibiotic resistance [44].

Our study emphasizes the importance of individual and group moral foundations that can inhibit the effect of believing in conspiracy theories on exhibiting health-related behaviors and policy support for health-protective measures. The indirect association of Beliefs in Conspiracy Theories with individual and group-based moral foundations and Policy Support, as well as containment health-related behaviors open novel interpretations of conspiracy beliefs as perceptions of facts, which are morally infused. Policy-making programs that aim to reduce the consistently negative impact of conspiracy beliefs on societal factors [45] such as adopting health-related behaviors and complying with policy measures could take into account that beliefs in conspiracy explanations of the origin and nature of the virus do not simply constitute antecedents of cognitive biases or failures, nor maladaptive behaviors based on personality traits. Instead, conspiracy beliefs as well as adoption of and support of policy measures reveal a deeper moral stance regarding what is right and/or wrong and who is to blame for the situation (e.g. the science, government, interest social groups etc.).

Beyond the contribution of our study, we should acknowledge some limitations of our study that could offer a fruitful avenue for future research. First, it is worth mentioning that while some of our effects were not very large, they might still have a big impact [46]. Specifically, as Abelson [47] has noted, seemingly smaller effect sizes can still matter in the long or shorter run. They can have the potential, when they are cumulated through longitudinal observations or observations across multiple countries or settings, to have important implications for psychological research. They can do so by offering explanations of life outcomes such as people’s public health, institutional trust and compliance, overall life quality and resilience against misinformation. Second, the study was correlational, thus it prevented us from inferring causations. Future research can inform existing findings by setting up experimental designs and testing the effects of moral traits on different forms of conspiracy theories and support for public health and other similar policies. Furthermore, speaking of a period when conspiracy theories have become widely attractive, an interesting avenue for future research could be whether people would be also inclined to endorse conspiracy theories about situations unrelated to COVID-19 events. For example, people who adopt a specific conspiracy explanation about COVID-19 as a hoax, may tend to describe other events (e.g., climate change, catastrophes, political or economic crises) in terms of conspiracies and hoaxes too in a context of a general conspiracy mentality, i.e. a tendency to broadly believe in conspiracy theories [48]. Because other events, unrelated to COVID-19, beg for collective actions and adoption of prescribed behaviors too (e.g., pro-environmental behaviors to protect the public and natural welfare) with moral underpinnings, it is of interest to identify similar relations between conspiracy beliefs, moral foundations and exhibition of actual prescribed behaviors. If we accept existing claims [49], suggesting a generalization of conspiracy beliefs about one specific event to other unrelated events, one could examine whether the respective effects of believing in conspiracy beliefs are also generalized to explain other events, as well.

### Policy-making impact of our research

Practitioners and policy makers can benefit from our research by acquiring information about people’s moral foundations that can mediate their inclination to endorse conspiracy beliefs with a subsequent effect on adopting necessary health-related behaviors to reduce the spread of the virus. In addition to this, national leaders can obtain useful information by this study, in order to get an overview of how they can effectively formulate and tailor political messages to appeal to people’s moral principles. Once people perceive political messages and decisions as moral ones, then they would be more likely to willingly adopt containing health-related behaviors and reject any conspiracy theories that explain the origin and spread of the virus. Indeed, people’s individual and collective health-related choices are put under moral constraints and raise important moral questions [50], potentially depending on what moral foundations politicians appeal to and whether they achieve to make people reject the conspiracy explanations of the origin and spread of the virus.

Tackling conspiracy information–known also with the popular term ‘infodemic’–during the COVID-19 pandemic, constitutes another one important task for governments and politicians. From a political communication perspective, conspiracy theories about COVID-19 often debunk political messages, distort public perception of the virus and, eventually, weaken the credibility of the source of such political information and guidelines [51]. Conspiracy theories about the pandemic have also reduced the perceived effectiveness of political messages and recommendations of health containment behaviors [52]. Thus, perceived morality constitutes an important dimension for policy-makers to take into account so that to reduce any distortion of information about the pandemic, enhancing public well-being and encouraging people’s engagement with health recommendations [53]. By considering the mediating role of morality–either in an individual or a group, cooperative level–in the association between beliefs in conspiracy theories about COVID-19 and the actual engagement with health containment behaviors and policy support, politicians can tailor their political messages to communicate strategically structured narratives advancing public health, appealing to individual and collective welfare ensuring, at the same time, the communication of comprehensive and accessible health information.

Overall, the COVID-19 pandemic constitutes a global challenge for governments and countries, as well as for the people whose lives have been significantly affected. In a digital era, when fake news, conspiracy theories and rumors circulate and spread impressively fast, the pandemic features an additional challenge: the identification of the actual effects of such conspiracies on important dimensions such as people’s moral judgements and behavioral change, factors which are crucial to determine the spread of the virus and the future of public health.

**Appendix A**: *Symbolization and Internalization Items of the Moral Identity Scale (Aquino & Reed*, *2002)*.

### Symbolization Items

- I often wear clothes that identify me as having these characteristics.
- The types of things I do in my spare time (e.g., hobbies) clearly identify me as having these characteristics.
- The kinds of books and magazines that I read identify me as having these characteristics.
- The fact that I have these characteristics is communicated to others by my membership in certain organizations.
- I am actively involved in activities that communicate to others that I have these characteristics.

### Internalization Items

- It would make me feel good to be a person who has these characteristics.
- Being someone who has these characteristics is an important part of who I am.
- I would be ashamed to be a person who had these characteristics.
- Having these characteristics is not really important to me.
- I strongly desire to have these characteristics.
